# Supplementary material for: Hexokinase-II Inhibition Synergistically Augments the Anti-tumor Efficacy of Sorafenib in Hepatocellular Carcinoma
Source: Int J Mol Sci. 2019 Mar 14;20(6):1292. doi: 10.3390/ijms20061292 (PMC6471302; doi:10.3390/ijms20061292)
Supplement: Supplementary file 1 [file ijms-20-01292-s001.zip › Supporting information_Yoo et al_20190307.docx]

**Supporting information**

**Sorafenib induces glycolytic pathway in hepatocellular carcinoma, which can be targeted by a hexokinase II inhibitor**

Jeong-Ju Yoo, Su Jong Yu, Juri Na, Juri Na, Kyungmin Kim, Young Youn Cho, Yun Bin Lee, Eun Ju Cho, Jeong-Hoon Lee, Yoon Jun Kim, Hey won Youn, Jung-Hwan Yoon

**Table of Contents**

1. **Supporting Materials and Methods……………………………………………..…….2**
2. **Supporting Figure Legends……………………………………………………………6**
3. **Supporting Tables……………………………………………………………………….7**
4. **References……………………………………………………………………………….20**

**Supporting Materials and Methods**

***Tumor growth kinetics***

To describe the tumor growth kinetics, an exponential model has been selected. Data were analyzed in the same manner as the previous study.(1, 2) The equation used after sorafenib and/or 3-BP administration was as follows: V=V0 × exp(k × T). V0 and V are the tumor volumes at baseline and T days later, and k is the growth rate constant related to the tumor doubling time.(1, 3) Data were analyzed with a nonlinear mixed effect modeling (NONMEM) software program (version V, level 1.1, Double Precision), and the first-order conditional estimation method and the PRED routine was used.(4) Exponential random effect models were used to model inter-individual variability for k and V0. For example, the baseline tumor volume was modeled as V0i = V0 × exp(ηi), where V0 indicates the typical value for baseline tumor volume for the population and V0i indicates the baseline tumor volume for an individual i. The ƞi is a random variable with normal distribution with mean 0 and variance ω2V0. Additionally, the combination of additive and proportional error model represented as Yij = Ŷij × (1 + εija) + εijp was used to model residual variability. In this model, Yij and Ŷij represent jth observed and predicted tumor volume in individual i, and εija and εijp are random variables of normal distribution with mean 0 and variances σa2 and σp2, for measurement j in individual i.

***Quantitation of apoptosis***

TUNEL assay by ApopTag In Situ Apoptosis Detectio Kits (Millipore) were performed to assess apoptosis in tumor tissue. Six high-power fields (×400) with randomly selected were investigated, and positive TUNEL cells were counted. The percentage of apoptotic cells were calculated by the ratio of apoptotic cells to total cells counted ×100. Minimum 400 cells were counted for each treatment. All procedures involving animals were approved by the Institutional Animal Care and Use Committee at Seoul National University, Republic of Korea and they were consistent with the Guide for the Care and Use of Laboratory Animals.

***Immunohistochemical analysis***

Anti-Hexokinase II (clone 3D3) and anti-Caspase 3 (clone ab13847) antibodies were purchased from Abcam. Anti-JNK (clone sc-6254) antibody was obtained from Santa Cruz Biotechnology. Immunostaining was done using Ventana Optiview system (Roche Diagnositics, Mannheim, Germany). Slides were scanned by Aperio ScanScope CS2 (Leica Biosystems, Nussloch, Germany) and image files of each core were obtained. PDI immunopositivity was calculated by the Positive Pixel Count Algorithm of the Aperio ImageScope (Leica Biosystems). Two or more cores per case were examined and the highest value was used as a representative value.

***Human study***

***Eligibility criteria, treatment regimen and assessment of response to sorafenib in patients with HCC***

The eligibility criteria for sorafenib therapy were (1) unresectable HCC according to the Barcelona clinic liver cancer (BCLC) staging classification (5, 6) (2) age < 80 years; (3) an Eastern Cooperative Group performance status of 0 or 1; (4) Child-Pugh grade A or B; (5) white blood cell count > 3,000 cells/mm^3^, hemoglobin level > 10 g/dL, platelet count >50,000 cells/mm^3^; and (6) serum total bilirubin < 3.0 mg/dL, serum transaminases < 200 IU/L and serum creatinine < 1.5 mg/dL. These eligibility criteria were based on the vulnerability to adverse side effects. The diagnosis of HCC was confirmed based on hematoxylin–eosin staining of histopathological specimens in all patients. Sorafenib was given orally at a dose of 400 mg twice daily. Treatment interruptions and up to two dose reductions (first to 400 mg once daily and then to 400 mg every 2 days) were permitted for drug-related adverse effects [the Common Terminology Criteria for Adverse Events (version 3)] (7). Treatment was continued until the radiologic progression, as defined by the modified Response Evaluation Criteria in Solid Tumors (mRECIST) (8). Assessed by contrast enhanced computed tomography or magnetic resonance imaging every 6-8 weeks, therapeutic response to sorafenib was defined according to the criteria of mRECIST.

***Immunohistochemical analysis***

Anti-Hexokinase II antibody (clone 3D3) for IHC was purchased from Abcam and immunostaining was done using Ventana Optiview system (Roche Diagnositics, Mannheim, Germany). Slides were scanned by Aperio ScanScope CS2 (Leica Biosystems, Nussloch, Germany) and image files of each core were obtained. PDI immunopositivity was calculated by the Positive Pixel Count Algorithm of the Aperio ImageScope (Leica Biosystems). Two or more cores per case were examined and the highest value was used as a representative value.

**Supporting Figure Legends**

**Figure S1. Effect of sorafenib and 3-BP on human HCC cell growth.** SNU-761 cells (A), and Huh-7 cells (B) were serum starved for 16 h and treated with sorafenib and/or 3-BP. Cell growth was determined using the MTS assay. n=3, Student’s t-test. **P* < 0.05

**Figure S2. Enhanced glycolysis after sorafenib could be targeted by a hexokinase II inhibitor.** SNU-761 cells were exposed to vehicle alone (control group), sorafenib alone (8 μM), 3-BP alone (75 μM) or a co-treatment of sorafenib (8 μM) + 3-BP (75 μM) for 3 hours. Extracellular lactate, the end-product of glycolysis, levels were measured by Lactate Colorimetric/Fluorometric Assay Kit (BioVision, CA, USA). n=3, Student’s t-test. **P* = 0.048.

**Figure S3. 3-BP improves the anti-tumor efficacy of sorafenib against orthotopic HCC tumors.** (A) SNU-761-luc hepatoma cells (5 × 105 / 20μL) were orthotopically implanted into the livers of recipient male BALB/c nu/nu mice. The establishment and growth of tumors were blindly monitored by bioluminescent imaging (BLI) by the Xenogen IVIS. The intensity of luciferase total flux signals, as measured by BLI represents the rate of proliferation. The mice were followed for up to 28 days. (B) The microscopy image of immunohistochemistry (TUNEL, anti-JNK, and anti-caspase 3)

**Figure S4. Upregulated intratumoral HK-II predicts poor survival in TCGA database.** Kaplan-Meier plots estimated overall survival in patients with HCC based on HK-II expression level. Median survival was 56.5 months and 84.7 months, respectively, for HK-II high and low group, which difference was also statistical significance (*P* = 0.041).

**Figure S5. Immunohistochemical analysis for HK-II protein expression in HCC patients cohort.** Representative images of high (left panel) and low (right panel) expression of HK-II. x 200.

**Supplementary Tables**

**Supplementary Table 1. Development of the tumor growth kinetics model by backward elimination from the full model**

| Hypothesis | -2 * log-likelihood | DF | Diff(-2 * log-likelihood) | Chi-square  (α=0.05) | p-value | Conclusion |
| --- | --- | --- | --- | --- | --- | --- |
| Base model |  |  |  |  |  |  |
| K value of each group was identical (k1=k2=k3=k4) | 5088.719 | 2 |  |  |  |  |
| Full model |  |  |  |  |  |  |
| Was k different according to treatment group? | 5055.716 | 5 | 32.463 | 7.81 (df=3) | <0.0001 | YES |
| Backward elimination from the Full model |  |  |  |  |  |  |
| Was k different between control group & sorafenib group? | 5057.109 | 4 | 1.393 | 3.84 (df=1) | 0.2379 | NO |
| Was k different between control group & 3-BP group? | 5069.281 | 4 | 13.565 | 3.84 (df=1) | <0.0001 | YES |
| Was k different between control group & sorafenib + 3-BP group? | 5066.877 | 4 | 11.161 | 3.84 (df=1) | <0.0001 | YES |
| Was k different between sorafenib group & 3-BP group? | 5076.960 | 4 | 21.244 | 3.84 (df=1) | <0.0001 | YES |
| Was k different between sorafenib group & sorafenib + 3-BP group? | 5073.222 | 4 | 17.506 | 3.84 (df=1) | <0.0001 | YES |
| Was k different between 3-BP group & sorafenib + 3-BP group? | 5055.750 | 4 | 0.034 | 3.84 (df=1) | 0.8537 | NO |

**Supplementary Table 2. Primer sequences for qRT-PCR**

| Primer name | Primer sequence |
| --- | --- |
| GADD153-for | 5´-TGAGCGTATCATGTTAAAGATGAGCG-3´ |
| GADD153-rev | 5´-GGTGTGGTGATGTATGAAGATACACTTCC-3´ |
| GADD34-for | 5´-TGATCCGGACCCTGAGACTCC-3´ |
| GADD34-rev | 5´-CCCAGACAGCCAGGAAATGG-3´ |

**Supplementary Table 3. Baseline Characteristics of study population in TCGA database**

| **Variable** | | **Total (*n*=224)** |
| --- | --- | --- |
|  |  |  |
| Age (years) (median (range)) | | 61 (17–85) |
|  | < 60 | 98 (43.8%) |
|  | ≥ 60 | 126 (56.2%) |
| Gender | |  |
|  | Male | 148 (66.1%) |
|  | Female | 76 (33.0%) |
| Etiology | |  |
|  | HBsAg positive | 53 (23.7%) |
|  | Anti-HCV positive | 19 (8.5%) |
|  | Alcohol | 64 (28.6%) |
|  | NAFLD | 7 (3.1%) |
|  | Hemochromatosis | 1 (0.4%) |
|  | Unknown | 80 (35.7%) |
| Child-Pugh score (median (range)) | | 5 (5–8) |
|  | A | 145 (64.7%) |
|  | B | 16 (7.1%) |
|  | Unknown | 63 (28.1%) |
| Alpha-fetoprotein (ng/mL) | |  |
|  | < 200 | 123 (54.9%) |
|  | ≥ 200 | 58 (25.9%) |
|  | Unknown | 43 (19.2%) |
| T stage | |  |
|  | T1 | 118 (52.7%) |
|  | T2 | 54 (24.1%) |
|  | T3 | 43 (19.2%) |
|  | T4 | 9 (4.0%) |
| N stage | |  |
|  | N0 | 162 (72.3%) |
|  | N1 | 3 (1.3%) |
|  | NX | 58 (25.9%) |
|  | Unknown | 1 (0.4%) |
| M stage | |  |
|  | M0 | 172 (76.8%) |
|  | M1 | 3 (1.3%) |
|  | MX | 49 (21.9%) |
| Resection type | |  |
|  | R0 | 199 (88.8%) |
|  | R1 | 10 (4.5%) |
|  | R2 | 1 (0.4%) |
|  | Rx | 10 (4.5%) |
|  | Unknown | 4 (1.8%) |
| Grade | |  |
|  | Grade 1 | 31 (13.8%) |
|  | Grade 2 | 100 (44.6%) |
|  | Grade 3 | 85 (37.9%) |
|  | Grade 4 | 6 (2.7%) |
|  | Unknown | 2 (0.9%) |

HBsAg, hepatitis B surface antigen; Anti-HCV, antibody against hepatitis C virus; NAFLD, nonalcoholic fatty liver disease.

**Supplementary Table 4. Baseline Characteristics of Study Population**

| **Variable** | | **Total (*n*=94)** |
| --- | --- | --- |
|  |  |  |
| Age (years) (median (range)) | | 54 (20–76) |
|  | < 60 | 61 (64.9%) |
|  | ≥ 60 | 33 (35.1%) |
| Gender | |  |
|  | Male | 82 (87.2%) |
|  | Female | 12 (12.8%) |
| Etiology | |  |
|  | HBsAg positive | 78 (82.1%) |
|  | Anti-HCV positive | 5 (5.3%) |
|  | Alcohol | 2 (2.1%) |
|  | Unknown | 10 (10.5%) |
| Child-Pugh score (median (range)) | | 5 (5–10) |
| Alpha-fetoprotein (ng/mL) | |  |
|  | < 200 | 53 (57.0%) |
|  | ≥ 200 | 40 (43.0%) |
| Tumor size | |  |
|  | < 5 cm | 86 (91.5%) |
|  | ≥ 5 cm | 8 (8.5%) |
| Tumor number | | 2.87 ± 3.54 |
| Vascular invasion | |  |
|  | No | 90 (95.7%) |
|  | Yes | 4 (4.3%) |
| Edmondson grade (worst) | |  |
|  | Grade 2 | 19 (20.2%) |
|  | Grade 3 | 35 (37.2%) |
|  | Grade 4 | 40 (42.6%) |
| HK-II expression | |  |
|  | Low | 22 (23.4%) |
|  | High | 72 (76.6%) |

PD, progressive disease; HBsAg, hepatitis B surface antigen; Anti-HCV, antibody against hepatitis C virus; HK, hexokinase.

**Supplementary Table 5. Factors identified on univariate and multivariate analyses that affect time to progression in HCC patients treated with sorafenib**

| **Variable** | | **Univariate Analysis** | |  | **Multivariate Analysis** | |
| --- | --- | --- | --- | --- | --- | --- |
|  |  | **HR** | ***P* Value^*^** |  | **Adjusted HR** | ***P* Value^*^** |
| Age (≥ 60 years) | | 0.559 (0.370–0.889) | 0.014 |  | 0.592 (0.373–0.937) | 0.026 |
| Male | | 0.908 (0.537–1.572) | 0.741 |  |  |  |
| Etiology | |  |  |  |  |  |
|  | anti-HCV positive versus HBsAg positive | 0.835 (0.261–2.669) | 0.761 |  |  |  |
|  | Alcohol versus HBsAg positive | 1.454 (0.528–4.032) | 0.478 |  |  |  |
|  | Unknown versus HBsAg positive | 0.631 (0.281–1.450) | 0.281 |  |  |  |
| Child-Pugh score | | 1.072 (0.958–1.170) | 0.196 |  |  |  |
| AFP (ng/mL) | |  |  |  |  |  |
|  | ≥ 200 | 1.114 (0.731–1.689) | 0.625 |  |  |  |
| Tumor size | |  |  |  |  |  |
|  | ≥ 5 cm | 1.208 (0.746–1.699) | 0.614 |  |  |  |
| Tumor number | | 1.031 (0.972–1.093) | 0.412 |  |  |  |
| Vascular invasion | |  |  |  |  |  |
|  | Yes | 1.493 (0.980–2.245) | 0.070 |  |  |  |
| Lymph node | |  |  |  |  |  |
|  | Yes | 1.579 (0.731–3.460) | 0.245 |  |  |  |
| Metastasis | |  |  |  |  |  |
|  | Yes | 2.521 (1.149–5.490) | 0.028 |  |  |  |
| Edmondson grade (worst) | |  |  |  |  |  |
|  | Grade 3 versus grade 2 | 0.735 (0.356–1.512) | 0.391 |  |  |  |
|  | Grade 4 versus grade 2 | 1.460 (0.721–3.011) | 0.312 |  |  |  |
| HK-II expression level | |  |  |  |  |  |
|  | High | 1.631 (1.032–2.590) | 0.039 |  | 1.909 (1.086–3.342) | 0.026 |

Abbreviations: Anti-HCV, antibody against hepatitis C virus; HBsAg, hepatitis B surface antigen; AFP, alpha-fetoprotein; HK, hexokinase.

**Supplementary Table 6. Factors identified on univariate and multivariate analyses that affect overall survival in HCC patients treated with sorafenib**

| **Variable** | | **Univariate Analysis** | |  | **Multivariate Analysis** | |
| --- | --- | --- | --- | --- | --- | --- |
|  |  | **HR** | ***P* Value^*^** |  | **Adjusted HR** | ***P* Value^*^** |
| Age (≥ 60 years) | | 0.820 (0.481–1.399) | 0.470 |  |  |  |
| Male | | 0.649 (0.309–1.363) | 0.265 |  |  |  |
| Etiology | |  |  |  |  |  |
|  | anti-HCV positive versus HBsAg positive | 2.049 (0.809–5.139) | 0.129 |  |  |  |
|  | Alcohol versus HBsAg positive | 0.764 (0.149–3.953) | 0.750 |  |  |  |
|  | Unknown versus HBsAg positive | 1.091 (0.123–9.390) | 0.941 |  |  |  |
| Child-Pugh score | | 2.114 (1.468–3.039) | <0.001 |  | 1.967 (1.348–2.871) | <0.001 |
| AFP (ng/mL) | |  |  |  |  |  |
|  | ≥ 200 | 1.155 (0.724–1.890) | 0.545 |  |  |  |
| Tumor size | |  |  |  |  |  |
|  | ≥ 5 cm | 1.345 (0.546–3.512) | 0.488 |  |  |  |
| Tumor number | | 1.112 (1.051–1.190) | 0.001 |  | 1.121 (1.033–1.189) | 0.002 |
| Vascular invasion | |  |  |  |  |  |
|  | Yes | 1.598 (0.731–3.532) | 0.253 |  |  |  |
| Lymph node | |  |  |  |  |  |
|  | Yes | 2.782 (1.521–5.021) | 0.002 |  | 2.142 (1.149–3.924) | 0.021 |
| Metastasis | |  |  |  |  |  |
|  | Yes | 1.366 (0.621–2.986) | 0.481 |  |  |  |
| Edmondson grade (worst) | |  |  |  |  |  |
|  | Grade 3 versus grade 2 | 1.323 (0.482–3.610) | 0.571 |  |  |  |
|  | Grade 4 versus grade 2 | 3.564 (1.391–9.221) | 0.008 |  |  |  |
| HK-II expression level | |  |  |  |  |  |
|  | High | 1.721 (1.072–2.912) | 0.039 |  | 1.882 (1.171–3.190) | 0.024 |

Abbreviations: Anti-HCV, antibody against hepatitis C virus; HBsAg, hepatitis B surface antigen; AFP, alpha-fetoprotein; HK, hexokinase.

**References**

1. Yu SJ, Yoon JH, Yang JI, Cho EJ, Kwak MS, Jang ES, Lee JH, et al. Enhancement of hexokinase II inhibitor-induced apoptosis in hepatocellular carcinoma cells via augmenting ER stress and anti-angiogenesis by protein disulfide isomerase inhibition. J Bioenerg Biomembr 2012;44:101-115.

2. Kwak MS, Yu SJ, Yoon JH, Lee SH, Lee SM, Lee JH, Kim YJ, et al. Synergistic anti-tumor efficacy of doxorubicin and flavopiridol in an in vivo hepatocellular carcinoma model. J Cancer Res Clin Oncol 2015;141:2037-2045.

3. Kim W, Yoon JH, Kim JR, Jang IJ, Bang YJ, Kim YJ, Lee HS. Synergistic anti-tumor efficacy of lovastatin and protein kinase C-beta inhibitor in hepatocellular carcinoma. Cancer Chemother Pharmacol 2009;64:497-507.

4. Beal SL. Population pharmacokinetic data and parameter estimation based on their first two statistical moments. Drug Metab Rev 1984;15:173-193.

5. Bruix J, Sherman M, American Association for the Study of Liver D. Management of hepatocellular carcinoma: an update. Hepatology 2011;53:1020-1022.

6. European Association For The Study Of The L, European Organisation For R, Treatment Of C. EASL-EORTC clinical practice guidelines: management of hepatocellular carcinoma. J Hepatol 2012;56:908-943.

7. DCTD N, NIH, DHHS. Cancer Therapy Evaluation Program, Common Terminology Criteria for Adverse Events (CTCAE). In. Version 3.0 ed; 2006.

8. Lencioni R, Llovet JM. Modified RECIST (mRECIST) assessment for hepatocellular carcinoma. Semin Liver Dis 2010;30:52-60.
