# Supplementary figures and images for: Hexokinase-II Inhibition Synergistically Augments the Anti-tumor Efficacy of Sorafenib in Hepatocellular Carcinoma
Source: Int J Mol Sci. 2019 Mar 14;20(6):1292. doi: 10.3390/ijms20061292 (PMC6471302; doi:10.3390/ijms20061292)

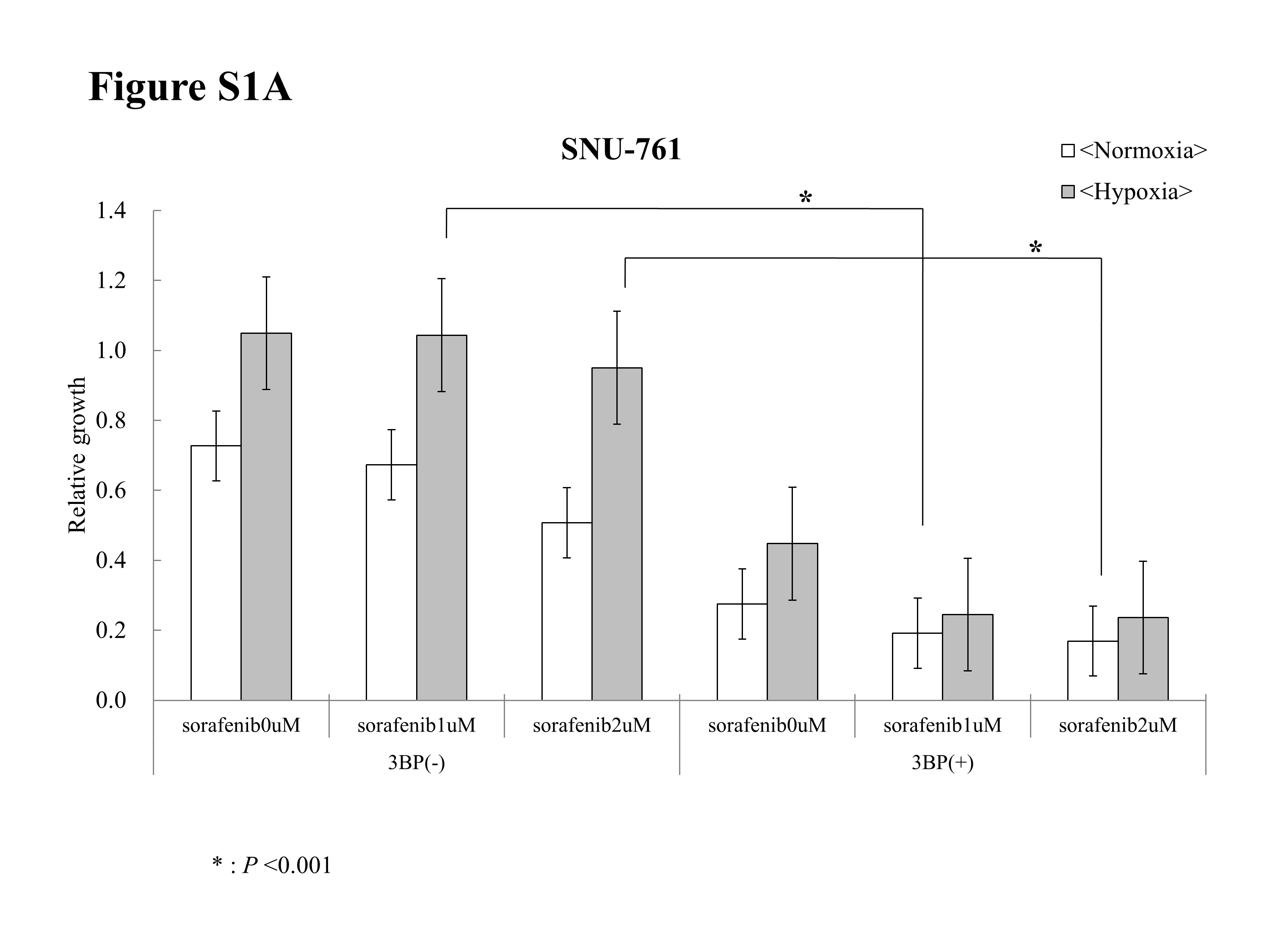

Supplement: Supplementary file 1 [file ijms-20-01292-s001.zip › Supplementary figures__Yoo et al_20190307_final/╜╜╢≤└╠╡σ1.TIF]

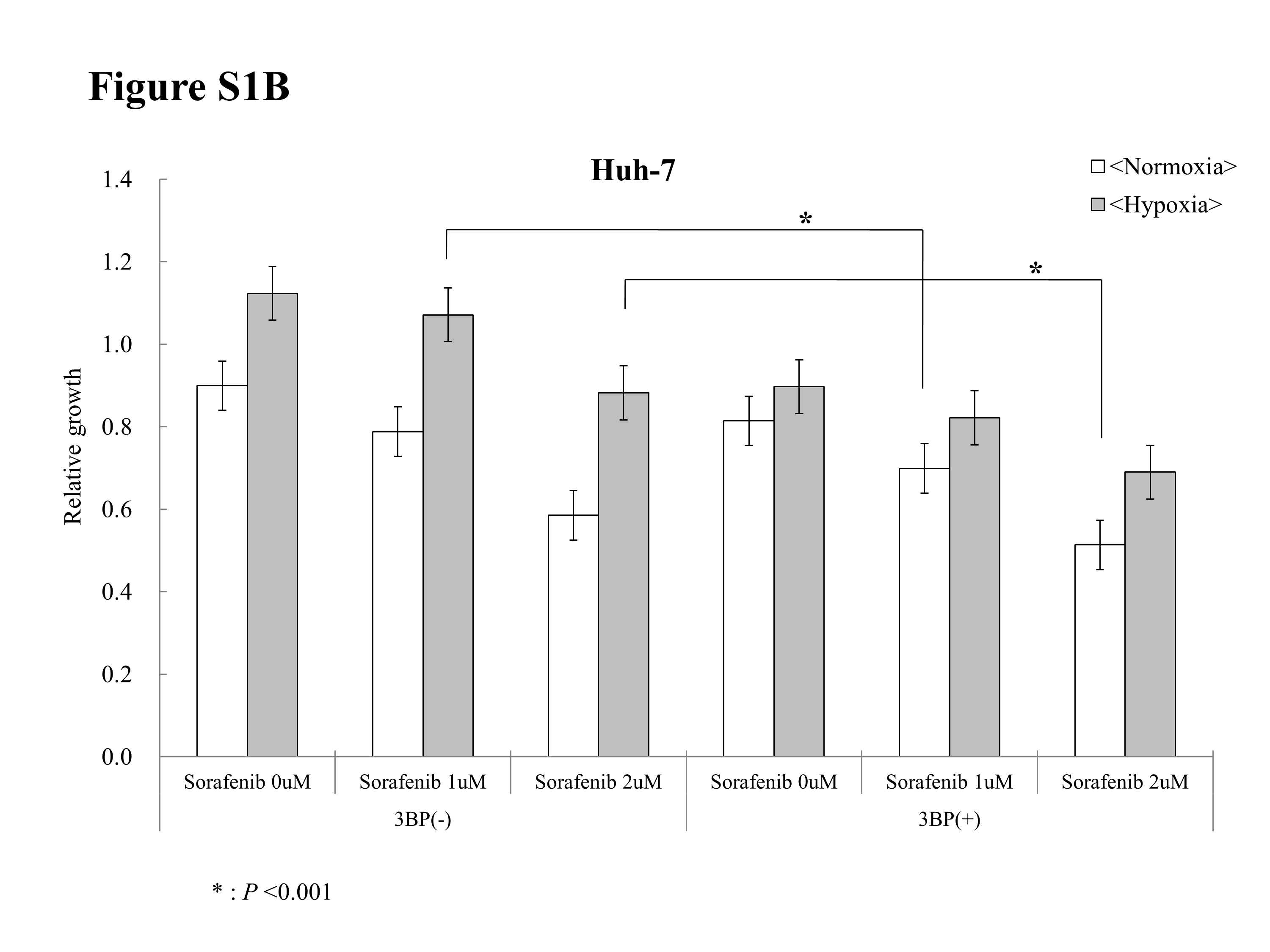

Supplement: Supplementary file 1 [file ijms-20-01292-s001.zip › Supplementary figures__Yoo et al_20190307_final/╜╜╢≤└╠╡σ2.TIF]

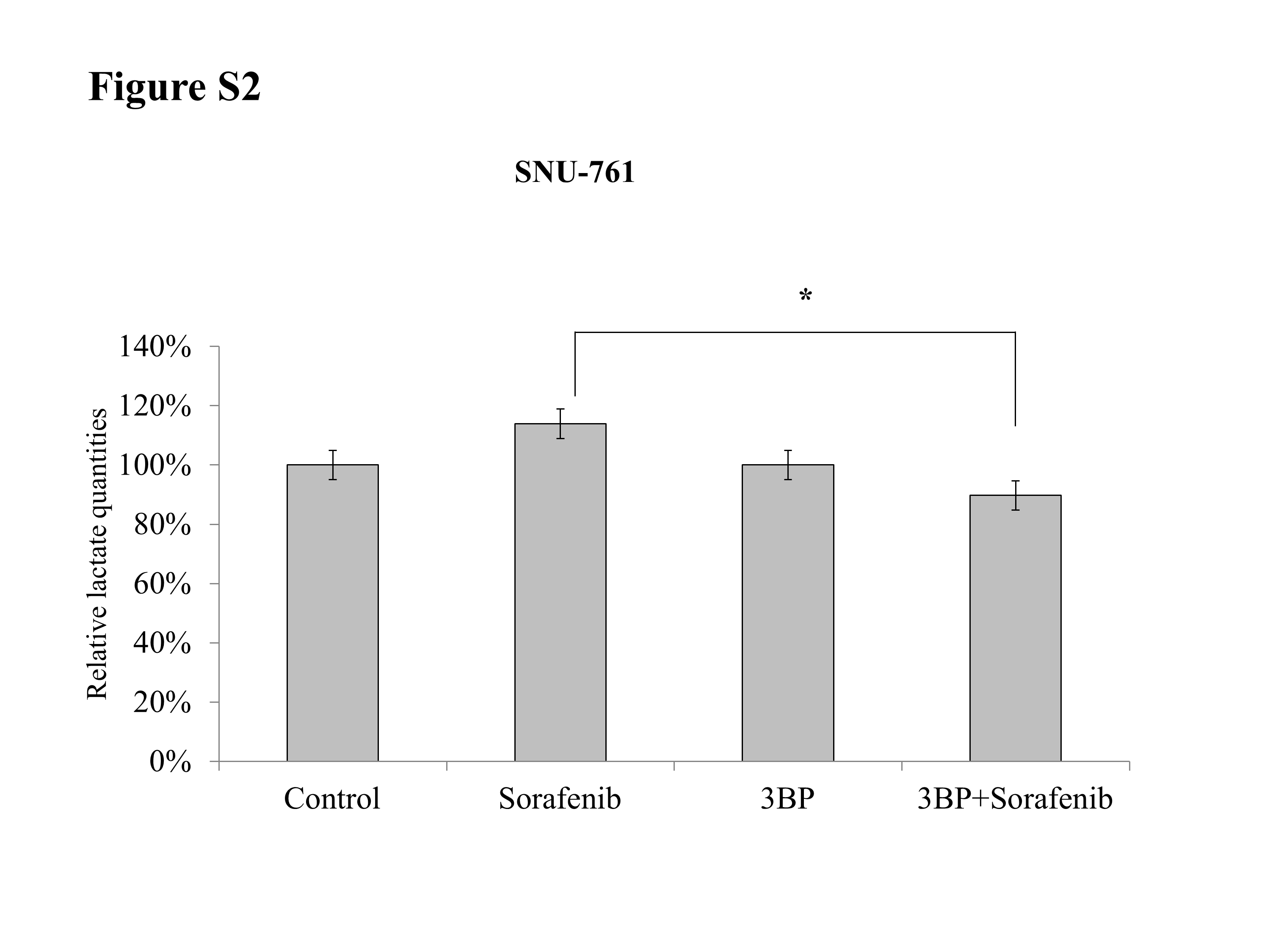

Supplement: Supplementary file 1 [file ijms-20-01292-s001.zip › Supplementary figures__Yoo et al_20190307_final/╜╜╢≤└╠╡σ3.TIF]

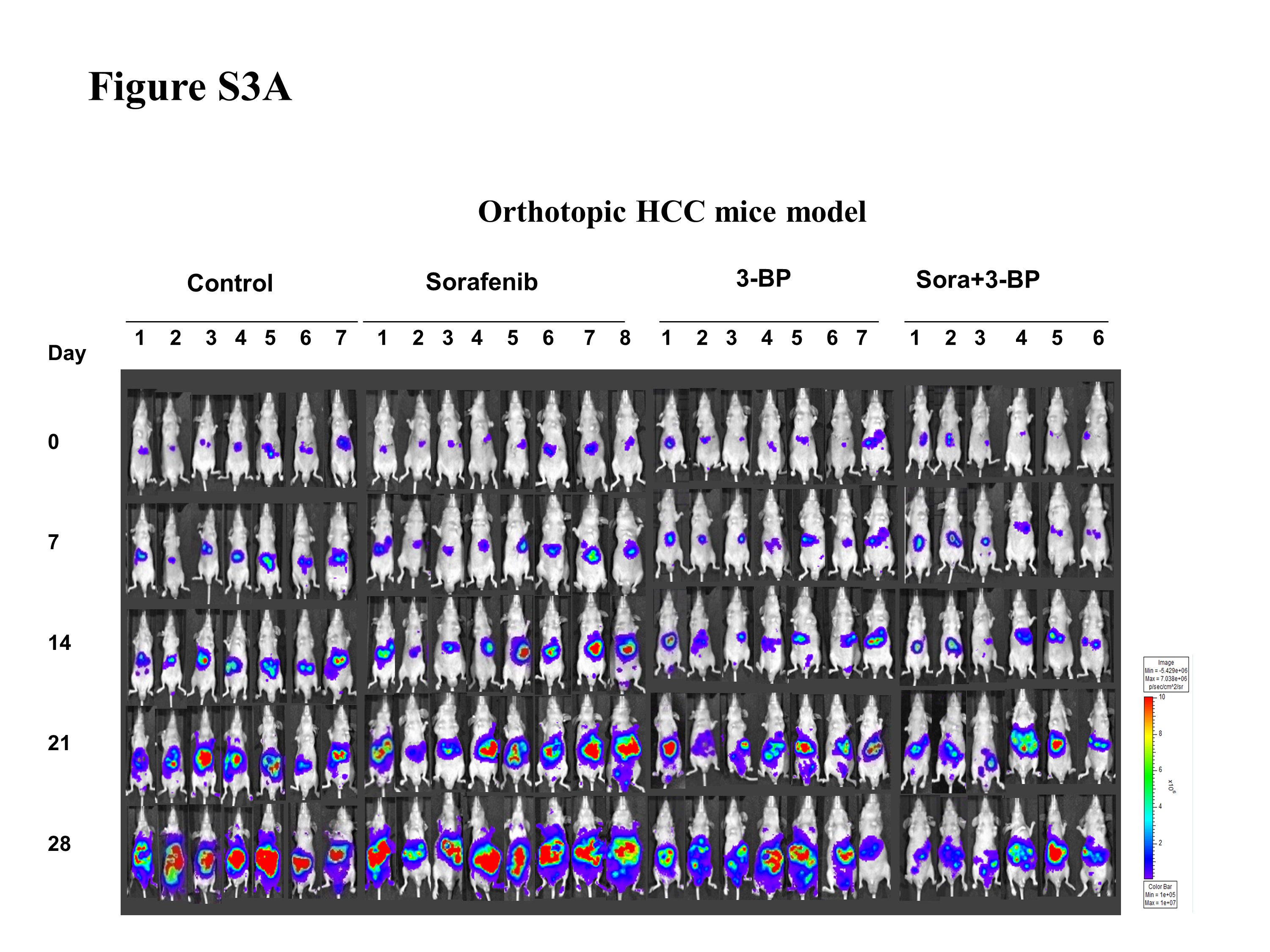

Supplement: Supplementary file 1 [file ijms-20-01292-s001.zip › Supplementary figures__Yoo et al_20190307_final/╜╜╢≤└╠╡σ4.TIF]

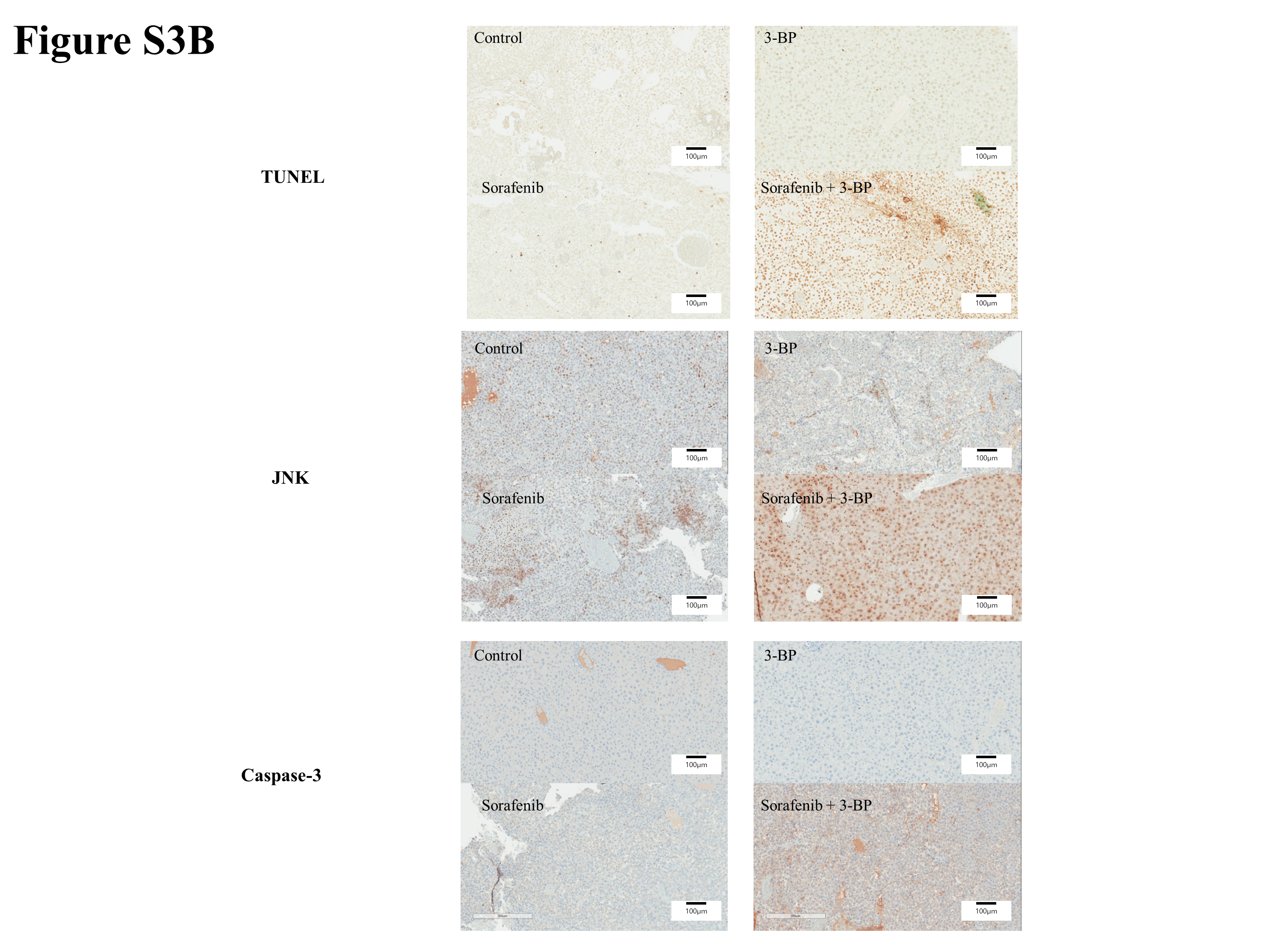

Supplement: Supplementary file 1 [file ijms-20-01292-s001.zip › Supplementary figures__Yoo et al_20190307_final/╜╜╢≤└╠╡σ5.TIF]

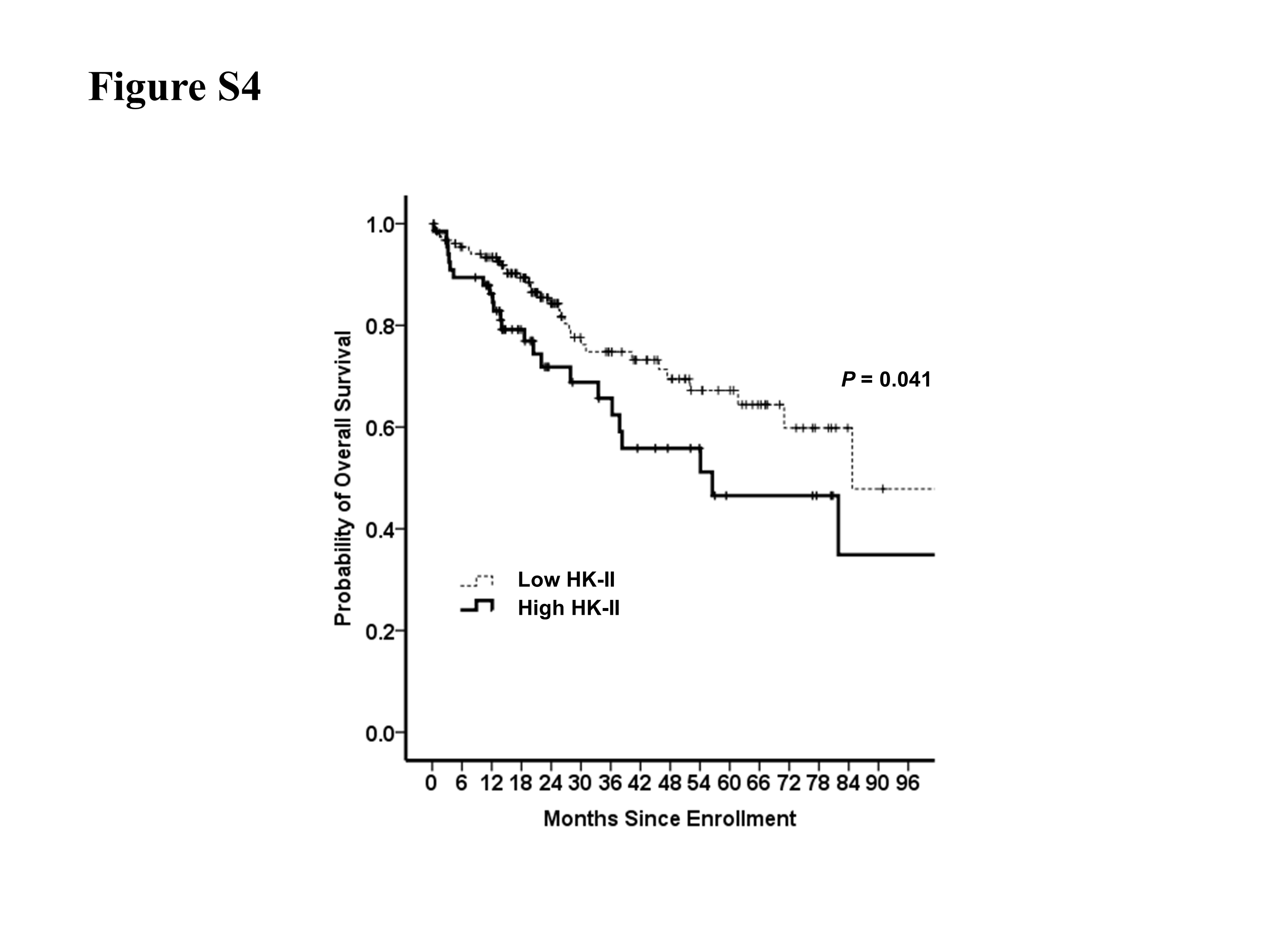

Supplement: Supplementary file 1 [file ijms-20-01292-s001.zip › Supplementary figures__Yoo et al_20190307_final/╜╜╢≤└╠╡σ6.TIF]

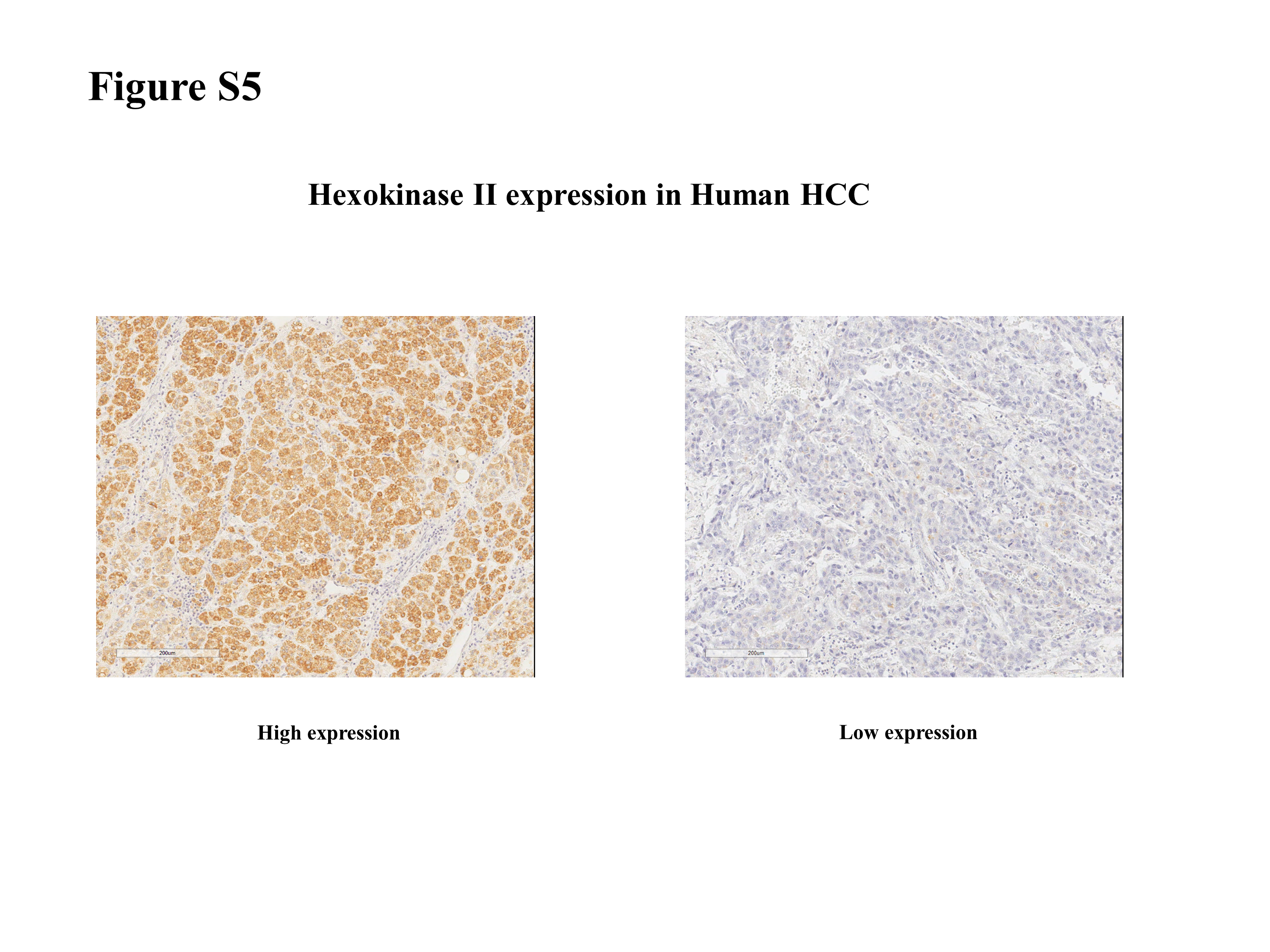

Supplement: Supplementary file 1 [file ijms-20-01292-s001.zip › Supplementary figures__Yoo et al_20190307_final/╜╜╢≤└╠╡σ7.TIF]
